# Relationship between dynamic changes of microorganisms in Qupi and the quality formation of Fengxiangxing Huairang Daqu

**DOI:** 10.3389/fmicb.2024.1435765

**Published:** 2024-07-08

**Authors:** Dan Cao, Jiali Lv, Jingying Chu, Shuangshuang Xu, Chengyong Jin, Yongli Zhang, Yuhang Zhang, Wen Zhang, Jie Kang

**Affiliations:** ^1^School of Food Science and Engineering, Shaanxi University of Science and Technology, Xi’an, China; ^2^Shaanxi Xifeng Liquor Co., Ltd., Baoji, China; ^3^Shaanxi Testing Institute of Product Quality Supervision, Xi’an, China

**Keywords:** Qupi changes, Daqu quality formation, microorganisms, microscopic characterization, fermentation characteristics, volatile metabolites, PacBio sequencing, HS-SPME-GC-MS

## Abstract

**Introduction:**

Fengxiangxing Huairang Daqu (FHD) is one of the major types of Daqu in China. However, the relationship between the microbial community structure at different stages, the changes in the sensory characteristics, fermentation characteristics, volatiles, the most critical process point, and the quality formation of FHD is not clear.

**Methods:**

Based on microscopic characterization, PacBio SMRT sequencing, and HS-SPME-GC-MS volatile metabolite analysis revealed the relationship between FHD quality formation and the dynamics of Qupi.

**Results:**

The results showed that the 12th day of the culture was the most critical process point, highlighting the most significant differences in microbial community structure, sensory characteristics, fermentation characteristics, and flavor substances. *Bacillus licheniformis* (43.25%), *Saccharopolyspora rectivirgula* (35.05%), *Thermoascus aurantiacus* (76.51%), *Aspergillus amstelodami* (10.81%), and *Saccharomycopsis fibuligera* (8.88%) were the dominant species in FHD. *S. fibuligera*, *A. amstelodami*, and *T. aurantiacus* were associated with the snow-white color of the FHD epidermis, the yellow color of the interior, and the gray-white color, respectively. The abundance of *T. aurantiacus*, *A. amstelodami*, *B. licheniformis*, and *S. rectivirgula* was positively associated with the esterifying power and liquefying power of FHD. The abundance of *T. aurantiacus* and *A. amstelodami* was positively correlated with the saccharifying power of FHD. The abundance of *S. fibuligera* was positively related to the fermenting power of FHD. A total of 248 volatiles were detected in Qupi, mainly including alcohols, esters, aldehydes, and ketones. Of them, eleven volatiles had a significant effect on the flavor of Qupi, such as 1-butanol-3-methyl-, hydrazinecarboxamide, ethanol, phenylethyl alcohol, ethyl acetate, 2-octanone, 1-octen-3-ol, formic acid-hexyl ester, (E)-2-octen-1-ol, ethyl hexanoate, and 2(3H)-furanone-dihydro-5-pentyl-. The abundance of *B. licheniformis*, *S. rectivirgula*, *T. aurantiacus*, and *S. fibuligera* was positively correlated with the alcohols, aromatic compounds, and phenols in FHD. The abundance of *S. fibuligera* was positively correlated with the acids, esters, and hydrocarbons in FHD.

**Discussion:**

These results indicate important theoretical basis and technical support for controllable adjustment of FHD microbial community structure, stable control of FHD quality, and precise, effective, and large-scale guidance of FHD production.

## 1 Introduction

Chinese liquor, brewed through a unique process using Daqu as the fermenting starter, is one of the six major distilled spirits in the world. Daqu is naturally formed by different raw materials under different temperatures, humidity, and other conditions. It contains abundant microbial community and enzyme system (Liu et al., 2018; Xia et al., 2022), which affects the quality of the Daqu and the aroma of the liquor (Zhou et al., 2021; Zhang et al., 2022a,b; Zhang Y. D. et al., 2022). For instance, different raw materials and their proportions, the shape of the Qupi, the geographical location of the fermentation room, environmental temperature and humidity, fermentation time of Daqu, etc., contribute to different aromas in Daqu, such as Qingxiangxing, Nongxiangxing, Jiangxiangxing, and Fengxiangxing (He et al., 2022; Zhang et al., 2023; Tang et al., 2024). Based on the maximum temperature the Qupi can reach during Daqu preparation, Daqu can be divided into high-temperature Daqu whose maximum temperature is 60°C–65°C, low-temperature Daqu whose maximum temperature is 40°C–50°C, and middle-temperature Daqu whose maximum temperature is 55°C–60°C (Fu et al., 2021; Yang L. et al., 2021; Yang Y. et al., 2021; Ding et al., 2022; Zhou et al., 2022). The process of transforming Qupi into Daqu involves three phases: the warming period, the high-temperature period, and the maturation period. Among them, the microbial community in the warming period is the richest; the thermophilic and heat-resistant microflora mainly survive and proliferate in the high-temperature period, and the heat-resistant microflora recover and reproduce in the maturation period (Fu et al., 2021; Ding et al., 2022; Huang et al., 2023). As such, the evolution of the microbial community in Daqu significantly affects and contributes to the dynamic changes in its fermentation characteristics and volatiles, thus affecting the quality of Daqu.

**Figure 1:**
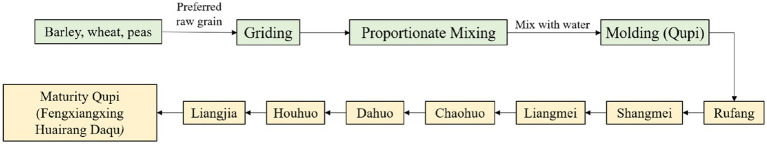
FHD preparation flow chart.

Fengxiangxing Daqu is one of the four major types of Daqu (Qingxiangxing, Nongxiangxing, Jiangxiangxing and Fengxiangxing) in China (Wei et al., 2020; Yu et al., 2024), mainly formed through a combination of the raw materials of Qingxiangxing Daqu and the medium-high-temperature production process of Nongxiangxing Daqu, showcasing the advantages of Nongxiangxing Daqu and Qingxiang Daqu with clear and rich aroma (Gou, 2022). The preparation of Fengxiangxing Huairang Daqu (FHD) consists of crushing and mixing barley, wheat, and peas with water, followed by mixing them well and then pressing them into bricks weighing about 1.5–4.5 kg and measuring 245 × 155 × 75 mm, which is called Qupi. Qupi was placed in the fermentation room for 30–35 days (Figure 1). Generally, Qupi preparation involves 9 stages, including molding Qupi, Rufang, Shangmei, Liangmei, Chaohuo, Dahuo, Houhuo, Liangjia, and maturity Qupi (FHD). During this process, the characteristics of Qupi closely related to the formation of FHD quality, such as sensory, microbial community, fermentation characteristics, and volatiles, underwent complex changes.

Studies on the community of Daqu and Qupi have reported that the signature bacterial community of Jiangxiangxing Daqu mainly includes *Kroppenstedtia*, *Bacillus*, and *Virgibacillus*, while the signature fungal community mainly includes *Thermoascus* and *Thermomyces* (Zhu et al., 2022). Similarly, the dominant bacterial community of Nongxiangxing Daqu mainly includes *Weissella*, *Lactobacillus*, and *Thermoactinomyces*, while the dominant fungal community mainly includes *Thermoascus, Pichia, Rhizopus, Rhizomucor*, and *Aspergillus* (He et al., 2022). The dominant bacterial community of Qingxiangxing Daqu mainly includes *Thermoactinomyces*, *Weissella*, *Lactobacillus*, *Staphylococcus*, and *Kroppenstedtia*, while the dominant fungal community mainly includes the genera *Thermomyces* (Quan et al., 2023). The dominant bacterial community of Fengxiangxing Daqu mainly includes *Lactobacillus*, *Weissella*, *Ruminococcus*, etc., and the dominant fungal community mainly includes *Thermoascus*, *Pichia, Aspergillus*, etc. (Zhang et al., 2022a; Zhang Y. D. et al., 2022). However, most of these studies were based on the sequencing of the V3-4 region of 16S rRNA of bacteria and the sequencing of the 1-5F region of ITS of fungi, which could only be annotated at the genus level but not at the species level. Moreover, these studies could not determine the specific dominant species, the evolution of the species during Daqu preparation, and their roles in the quality formation of Daqu. Therefore, it is difficult to accurately guide the production of Daqu. In recent years, the highly organized strain annotation has provided the basis for in-depth analysis of the species composition of the samples (Hou et al., 2022). Compared with the second-generation short fragment sequencing, the third-generation sequencing technology represented by the single-molecule real-time sequencing technology of Pacific BioSciences has the advantages of long read lengths and high throughput. The results of repeated sequencing of the same sequence can be corrected for each other through the unique circular consensus sequencing mode, thus obtaining consistent sequences with very high sequencing quality (Yang et al., 2022). Moreover, researchers can obtain species-level species composition and better resolution of species annotation further using this technique. This technique has also been applied in the research of Daqu (Zhang et al., 2022a; Zhang Y. D. et al., 2022; Han et al., 2023). However, in-depth analysis of the primary functional microbial species in the Fengxiangxing Daqu system is still scarce.

In the studies of the fermentation characteristics and metabolites of Daqu and Qupi and their correlations, the recent development of the HS-SPME-GC-MS technology has allowed for maximum detection of volatiles related to the flavor quality of Qupi, playing an important role in analyzing and guiding the production of Daqu (Fan et al., 2019; Cai et al., 2022; Yang et al., 2022; Zhang et al., 2022b; Zhang Y. D. et al., 2022; Dong et al., 2024). For instance, Jiangxiangxing Daqu has stronger ability to produce aroma and ester, and the volatile metabolites are more complex with relatively high content of aldehydes and pyrazines (Yi et al., 2019; Cai et al., 2021); Nongxiangxing Daqu has higher content of ethyl esters (He et al., 2022; Xia et al., 2022; Quan et al., 2023); Qingxiangxing Daqu has higher saccharifying and fermenting power and weaker aroma-producing ability (Zheng et al., 2014; Quan et al., 2023). Moreover, the high-temperature period is mainly associated with volatile metabolites production, which has a guiding effect on the quality formation of Daqu and is the key to determining the aroma type of Daqu and baijiu (Yi et al., 2019). However, most of these studies were based on the structure, fermentation characteristics, and volatiles and their correlations with macroalgae at the genus level, making it difficult to accurately investigate the influence of microbial species on the quality formation of Daqu.

Research on the relationship between the dynamic changes in Qupi, including the changes in microbial community structure at different stages, the changes in the sensory characteristics, fermentation characteristics, volatiles, the most critical process point, and the quality formation of FHD is not clear. Therefore, the present study aimed to analyze the microbial community structure, succession pattern, sensory characteristics, fermentation characteristics, and volatile metabolites during the transformation of Qupi into Daqu and explore their relationship with the quality formation of FHD using stereo microscopy, PacBio SMRT, and headspace solid-phase microextraction and gas chromatogyraphy-mass spectrometry (HS-SPME-GC-MS). Combined with the fermentation characteristics, this study provided scientific insights into the quality formation of FHD. The dominant microorganisms in FHD were explored through dynamic analysis, which provided a reference for further research on the quality formation mechanism of FHD. Overall, these results provide a theoretical basis and technical support for controllable adjustment of FHD microbial community structure, stable control of FHD quality, and precise, effective, and large-scale guidance of FHD production.

## 2 Materials and methods

### 2.1 Sample collection

**Figure 2:**
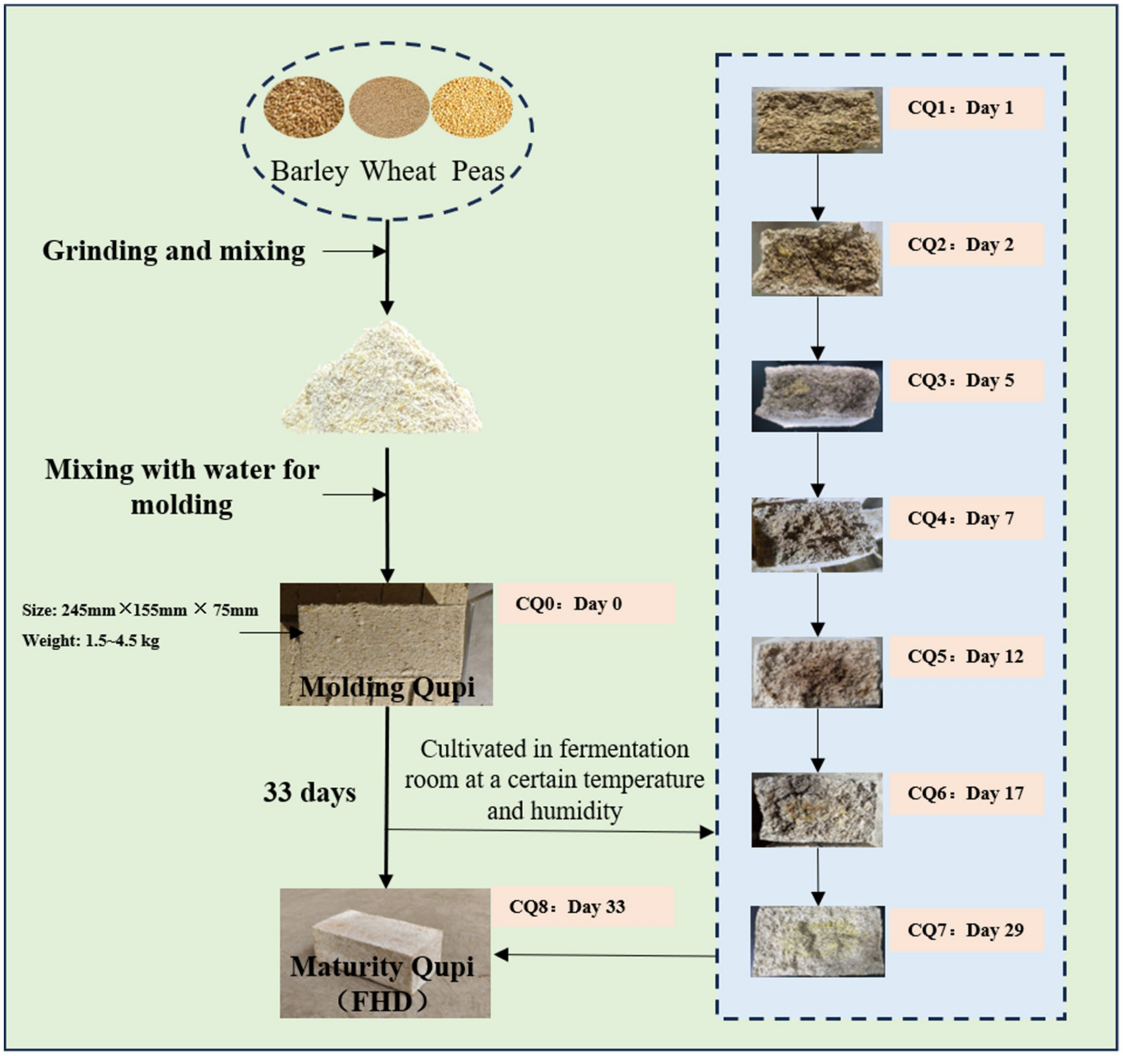
Test sample collection chart.

FHD sampling was carried out in the Daqu workshop of Shaanxi Xifeng Liquor Co. in Liulin Town, Baoji City, Shaanxi Province, China. The nine points of dynamic changes in Qupi during the forming FHD from Qupi, as shown in Figure 2, marked as CQ0, CQ1, CQ2, CQ3, CQ4, CQ5, CQ6, CQ7, CQ8, respectively. The samples were taken separately according to the five-point method and then crushed and mixed well immediately after removal. Later, the sample was sealed in sterile self-sealing bags and stored at −80°C for fermentation characterization, microbial diversity, and volatile matter analysis.

### 2.2 Stereomicroscopic characterization

The microscopical surface and the interior of the Qupi at each of the nine points during the forming FHD from Qupi were characterized through a LeicaS9i stereo microscope to obtain typical images.

### 2.3 Fermentation characterization

The fermentation characteristics of each Qupi sample were determined in triplicate using standard methods. Acidity, starch content, moisture, fermenting power, saccharifying power, esterifying power, and liquefying power were determined based on the national professional standard techniques (QB/T 4257–2011) (Ministry of Industry and Information Technology of the People’s Republic of China, 2012).

### 2.4 Microbial diversity analysis

#### 2.4.1 DNA extraction and PCR amplification

Microbial community genomic DNA was extracted from Qupi samples using the Genomic DNA Extraction Kit (QIAGEN, Duesseldorf, Germany) according to manufacturer’s instructions. The DNA extract was checked on 1% agarose gel, and DNA concentration and purity were determined with NanoDrop 2000 UV–vis spectrophotometer (Thermo Scientific, Wilmington, United States). For bacterial community, the bacterial 16S rRNA genes were amplified using the universal bacterial primers 27F (5′-AGRGTTYGATYMTGGCTCAG-3′) and 1492R (5′-RGYTACCTTGTTACGACTT-3′) (Weisburg et al., 1991). For fungi community, the ITS sequences were amplified using the primers ITS1F (5′-CTTGGTCATTTAGAGGAAGTAA-3′) and ITS4R (5′-TCCTCCGCTTATTGATATGC-3′) (Souza et al., 2014). Primers were tailed with PacBio barcode sequences to distinguish each sample. Amplification reactions (20-μL volume) consisted of 5 × FastPfu buffer 4 μL, 2.5 mM dNTPs 2 μL, forward primer (5 μM) 0.8 μL, reverse primer (5 μM) 0.8 μL, FastPfu DNA Polymerase 0.4 μL, template DNA 10 ng and DNase-free water. The PCR amplification was performed as follows: initial denaturation at 95°C for 3 min, followed by 27 cycles of denaturing at 95°C for 30 s, annealing at 60°C for 30 s and extension at 72°Cfor 45 s, and single extension at 72°C for 10 min, and end at 4°C [T100 Thermal Cycler (BIO-RAD, United States)]. PCR reactions were performed in triplicate. After electrophoresis, The PCR products were purified using the AMPure^®^ PB beads (Pacifc Biosciences, CA, United States) and quantified with Synergy HTX (Biotek, United States).

#### 2.4.2 DNA library construction and sequencing

Purified products were pooled in equimolar and DNA library was constructed using the SMRTbell^®^ prep kit 3.0 (Pacifc Biosciences, CA, United States) according to PacBio’s instructions. Purified SMRTbell libraries were sequenced on the Pacbio Sequel IIe System (Pacifc Biosciences, CA, United States) by Majorbio Bio-Pharm Technology Co. Ltd. (Shanghai, China).

#### 2.4.3 Data processing and statistical analysis

PacBio raw reads were processed using the SMRTLink analysis software (version 11.0) to obtain high-quality Hifi reads with a minimum of three full passes and 99% sequence accuracy. Hifi reads were barcode-identified and length-filtered. For bacterial 16S rRNA gene, sequences with a length < 1,000 or > 1,800 bp were removed. For fungi ITS, sequences with a length < 300 bp or > 900 bp were removed.

The Hifi reads were clustered into operational taxonomic units (OTUs) using USEARCH 11 (Stackebrandt and Goebel, 1994; Edgar Robert, 2013) with 97% sequence similarity level. The most abundant sequence for each OTU was selected as a representative sequence. To minimize the effects of sequencing depth on alpha and beta diversity measure, the number of 16S rRNA gene sequences from each sample were rarefied to 6,000, which still yielded an average Good’s coverage of 99.09%, respectively. The taxonomy of each OTU representative sequence was analyzed by RDP Classifier version 2.13 (Wang, 2007) against the NT_ 16 s (v20221012)/UNITE _ITS (8.0) database using confidence threshold of 0.7.

Bioinformatic analysis of the Qupi microbiota was carried out using the Majorbio Cloud platform.^1^ Based on the OTUs information, rarefaction curves and alpha diversity indices including ace, chao, shannon, simpson, coverage, and sobs indices were calculated with Mothur v1.30.2. The similarity among the microbial communities in different samples was determined by principal coordinate analysis (PCoA) based on Bray–curtis dissimilarity using Vegan v2.4.3 package. The linear discriminant analysis (LDA) effect size (LEfSe)^2^ was performed to identify the significantly abundant taxa of species among the different groups (Segata et al., 2011). Spearman’s rank correlations between Qupi microbial species and were calculated to reveal the relationships among microbial communities. Only significant correlations (*p* < 0.05, with false discovery rate correction) were considered as valid correlations. The redundancy analysis (RDA) was performed using Vegan v2.4.3 package to investigate effect of environmental factors on Qupi microbial community structure. Values of the x- and y-axis and the length of the corresponding arrows represented the importance of each environmental factors in explaining the distribution of taxon across communities. Correlations between microbial communities and major environmental factors in different samples were determined based on Spearman’s rank correlations using Vegan v3.3.1 package. The internal community relationships across the samples were explored by plotting correlation heat maps (Barberan et al., 2012). The relevant box-plot maps were generated using Origin 2019. * denotes *p* < 0.05, ** denotes *p* < 0.01, and *** denotes *p* < 0.001.

### 2.5 Volatile metabolites analysis

#### 2.5.1 HS-SPME-GC-MS analysis

Qupi samples were harvested, weighted, immediately frozen in liquid nitrogen, and stored at −80°C until needed. The samples were analyzed using a Shimadazu 2030 GC equipped with a QP2020 NX mass spectrometry system (Shimadazu, Tokyo, Japan). In this process, 2.0 g of Qupi powder within a 20 mL head-space vial, then internal 10 μL 2-octanol (50 μg/mL) were added. After that, the volatile metabolites in mixture were extracted by an automatic headspace sampler system (AOC-6000) at 50°C for 30 min. Subsequently, the SPME fiber (DVB/C-WR/PDMS) was introduced into the injection port, set at 250°C, for a duration of 5 min (Huang et al., 2023). Metabolites separation was achieved using a Shimadazu SK-MAX column (30 m × 0.25 mm; 0.25 μm film thickness), and followed by analysis via GC–MS analysis system. Helium was used as the carrier gas at a linear velocity of 3.0 mL/min. The oven temperature was programmed from 40°C (3.5 min), increasing at 4°C/min to 230°C, hold for 6 min. Mass spectra was recorded in electron impact (EI) ionization mode at 70 eV. The MS was selected ion monitoring (SIM) mode was used for the identification and quantification of analytes. At least 5 replicates were done for each sample (Liu et al., 2022).

#### 2.5.2 Data processing and statistical analysis

The raw data of GC/MS is preprocessed by GC–MS solution 4.52. Metabolites identification was carried out by comparing mass spectral profiles with a match quality of ≥80 in the NIST 20.0 database, the volatiles of the samples were quantified by the internal standard method. After which the metabolites were searched and classified using the HMDB^3^ database. The R package (version 3.5.1) performed principal component analysis (PCA). In addition, student’s *t*-test and fold difference analysis were performed. The selection of significantly different metabolites was determined based on the variable importance in the projection (VIP) obtained by the PLS-DA model and the *p*-value of student’s t test. The metabolite quantity and classification changes were visualized by Origin 2019, R package (version 4.2.0), and R package (version 3.5.1). The biochemical pathways were mapped through metabolic enrichment and pathway analysis based on database search (KEGG).^4^

## 3 Results

### 3.1 Dynamic changes in microscopic features

**Figure 3:**
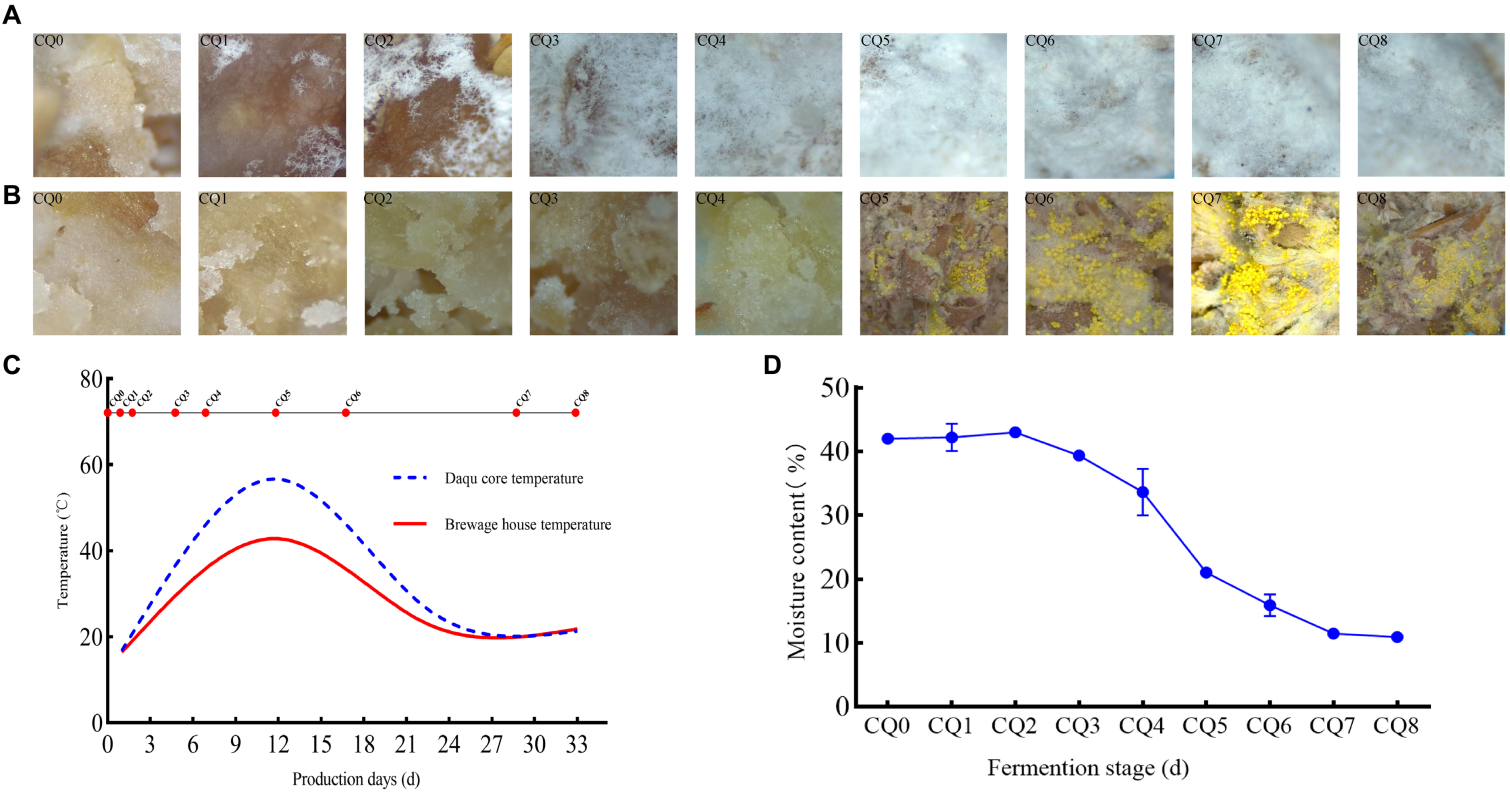
Dynamic changes in surface micro-characterization **(A)**, internal micro-characterization **(B)**, temperature **(C)**, moisture content **(D)** of Qupi at nine points during the forming FHD from Qupi.

The somatic microscopy characterization of the surface and interior of the Qupi at each of the nine points during the forming FHD from Qupi showed (Figures 3A,B) the most significant differences in filamentous microbial changes between the surface and interior of the Qupi. From CQ0 to CQ8, the snowflake-like white mycelium on the surface of Qupi increased with the quenching time, and the whole surface of Qupi was gradually covered by white mycelium on the 12th day (CQ5), which tended to be stabilized. It showed that the filamentous microorganisms with snowflake-like white mycelium grew vigorously before CQ5 (≤45°C) and stopped growing when the temperature reached the highest (55°C–60°C) at the CQ5 stage. Moreover, the mycelium was not full after CQ5 (≤45°C), showing the aging morphology; before the CQ5 stage, the growth of distinguishable microorganisms did not appear inside the Qupi, but a lot of seemingly granular materials were observed, which might be the microflora colony in the germination state. After CQ5, the gray-white mycelium and filamentous microorganisms with bright yellow ascospores grew vigorously, and the concentration of the microflora increased sharply to reach the maximum when the Qupi was mature. This result showed that the surface of Qupi was suitable for its growth due to sufficient oxygen and temperature close to room temperature; thus, the growth rate of filamentous microorganisms was significantly faster than others, which was conducive to preventing the invasion of harmful fungus. The growth of mycelium was stopped and stabilized due to the high temperature at CQ5 (12 days, 55°C–60°C). Compared with the surface, the inside of the Qupi was slow due to insufficient oxygen and high temperature. The filamentous microorganisms could not be detected at CQ5, suggesting that the filamentous microorganisms were mainly thermophilic or heat-resistant microflora. In conclusion, CQ5 (12 days, 55°C–60°C) was considered the key time point for FHD preparation; the community changes were most significant, and temperature and humidity were the main factors affecting the growth of the community.

### 3.2 Temperature and moisture dynamics

The forming FHD from Qupi lasted for 30–35 days, which can be divided into three periods according to the temperature changes: warming period I (CQ0–CQ3, 20°C–45°C), high-temperature period II (CQ4–CQ5, 45°C–60°C), and maturation period III (CQ6–CQ8, 60°C–20°C) (Figure 3C). The temperature changes inside the Qupi were higher at the nine points (18°C–60°C), while the temperature changes in the fermentation room were lower (18°C–40°C). The moisture content of CQ0, CQ1, CQ2, CQ3, CQ4, CQ5, CQ6, CQ7, and CQ8 was 41.986, 42.211, 42.987, 39.368, 33.618, 21.063, 15.933, 11.473, and 10.933%, respectively, which showed a decreasing trend (Figure 3D). Among them, CQ0 had the highest moisture content (41.986 ± 0.250%), CQ8 had the lowest moisture content (10.933 ± 0.375%), and CQ5 had the greatest change in moisture before and after the fire. This indicates that CQ5 is a critical point for both temperature and moisture changes, and temperature and moisture are the most important factors affecting microbial growth.

### 3.3 Dynamics of fermentation characteristics

The results of the fermentation characteristics of Qupi at nine points during the forming FHD from Qupi are shown in Table 1. The residual starch content decreased gradually from 62.483 ± 0.371% in CQ0 to 50.580 ± 0.06% in CQ8, and the difference between the stages was insignificant, indicating that the microflora did not have a strong ability to detoxify starch. The esterifying power showed a gradual upward trend, with the lowest being observed in CQ0 (23.167 ± 4.637 U) and the highest in CQ6 (591.333 ± 7.345 U). The esterifying power greatly increased from CQ3 to CQ6 (591.333 ± 7.345 U) and reached the highest in CQ7–CQ8 and CQ6, but the change was not significant, indicating that high temperature is conducive to esterification reaction. Acidity reached the highest (2.134 ± 0.010 mmol/10 g) in CQ0 and lowest (0.570 ± 0.010 mmol/10 g) in CQ8, showing fluctuating changes in the middle, but the overall acid production capacity was not strong, which is also the quality of the maturation period of Qupi quality requirements. The liquefying power showed an increasing trend, with the highest after CQ5 and CQ8 (0.686 ± 0.010 U), indicating that high temperature is favorable to the liquefaction of starch. The saccharifying power was higher in CQ0–CQ2, followed by CQ6–CQ8, and lower in CQ3–CQ5, with the highest saccharifying power in CQ0 (717.667 ± 9.074 U) and the lowest in CQ5 (249.083 ± 3.033 U), indicating that high temperature is unfavorable to the saccharification of starch. The fermenting power was higher in CQ2–CQ4, with the highest in CQ4 (1.376 ± 0.094 U), which was higher than the warming and maturation periods. Generally, yeast has a stronger fermentation ability, but it takes a certain amount of time to grow, and most of them are intolerant of high temperatures, so the peak of the fermenting power appeared in CQ2. In conclusion, the different temperatures and moisture contents in the Qupi at the nine points affected the growth and self-assembly of the microflora, thereby affecting the fermentation characteristics of Qupi.

**Table 1:** Fermentation characteristics of Qupi during the forming FHD from Qupi.

Stage	Acidity/(mmol·10 g^−1^)	Starch content/%	Esterifying power/U	Liquefying power/U	Saccharifying power/U	Fermenting power/U
CQ0	2.173 ± 0.04	62.483 ± 0.30	23.167 ± 3.79	0	717.667 ± 7.41	0.423 ± 0.08
CQ1	1.877 ± 0.05	61.133 ± 0.29	39.367 ± 6.53	0	733.333 ± 13.70	0.122 ± 0.05
CQ2	1.485 ± 0.09	59.947 ± 0.28	89.733 ± 7.21	0	697.333 ± 4.11	1.300 ± 0.05
CQ3	0.615 ± 0.05	62.233 ± 0.30	101.633 ± 1.23	0.261 ± 0.04	271.667 ± 14.88	1.316 ± 0.07
CQ4	0.930 ± 0.04	58.327 ± 0.12	176.600 ± 8.68	0.256 ± 0.02	249.000 ± 2.45	1.376 ± 0.08
CQ5	1.100 ± 0.05	56.173 ± 0.15	241.186 ± 16.08	0.183 ± 0.01	341.333 ± 0.94	0.213 ± 0.05
CQ6	0.759 ± 0.10	55.332 ± 0.15	591.333 ± 6.03	0.434 ± 0.01	423.000 ± 46.73	0.574 ± 0.16
CQ7	1.045 ± 0.05	51.967 ± 0.49	547.703 ± 4.11	0.281 ± 0.02	430.083 ± 20.45	0.336 ± 0.03
CQ8	0.570 ± 0.01	50.580 ± 0.05	568.061 ± 2.37	0.679 ± 0.01	574.000 ± 11.31	0.870 ± 0.06

### 3.4 Dynamics of microbial communities

#### 3.4.1 Changes in microbial diversity indices

**Figure 4:**
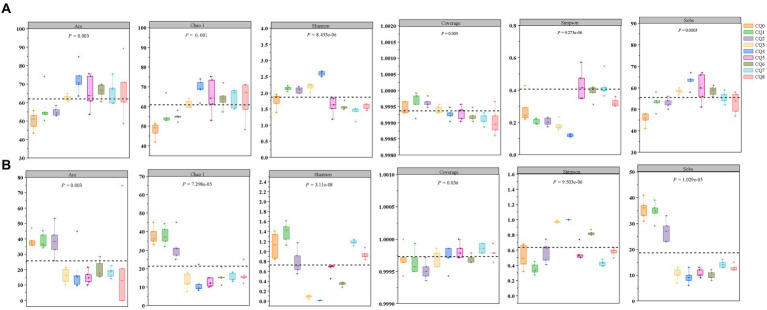
Changes in the α-diversity indices of bacteria **(A)** and fungi **(B)** at nine points of Qupi during the forming FHD from Qupi.

A total of 993,293 and 1,065,404 fungi optimized sequences were obtained from 45 samples through PacBio sequencing of the microbial community of Qupi during the forming FHD from Qupi. Six α-diversity indices, Ace, Chao, Shannon, Simpson, Coverage, and Sobs, were utilized to reflect the richness and diversity of the microbial communities in the samples (Figure 4). As shown in Figure 4A, the overall bacterial diversity during the forming FHD from Qupi showed an increase, followed by a decreasing trend. Both Shannon and Simpson indices indicated that the bacterial diversity was low in CQ7, with the highest in CQ3 and CQ4, and the diversity in the CQ4 stage was significantly different from that in the other eight stages. The Sobs, Chao, and Ace indices indicated that the bacterial species richness was higher in the high-temperature period than in the warming and maturation periods.

Figure 4B shows the overall fluctuating trend of fungi diversity during the forming FHD from Qupi. The Shannon and Simpson indices showed that the fungi diversity reached the highest in the CQ1 stage and significantly decreased to the lowest when the preparation temperature reached the highest. The Sobs, Chao, and Ace indices all showed that the species richness was higher in the CQ1–CQ2 stage, which significantly decreased in the CQ3–CQ5 stage, with a small increase in maturity. Additionally, the Coverage index was all higher than 0.99, indicating that the data can truly reflect the composition and distribution of Qupi microflora during the forming FHD from Qupi and are valid.

#### 3.4.2 Changes in microbial community structure

**Figure 5:**
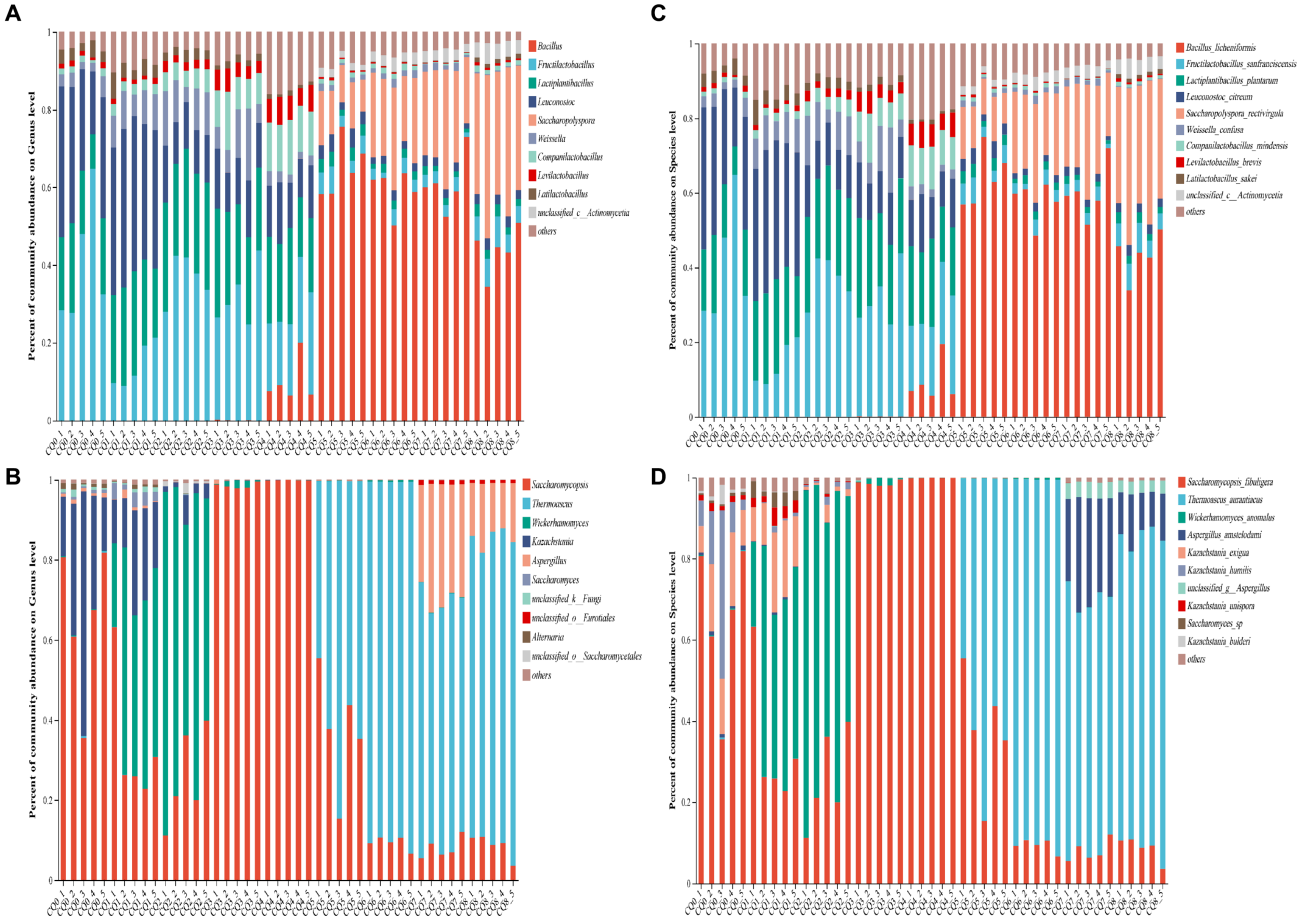
Community composition at the bacterial and fungi genus level **(A,B)** and species level **(C,D)** in Qupi at nine points during the forming FHD from Qupi.

**Figure 6:**
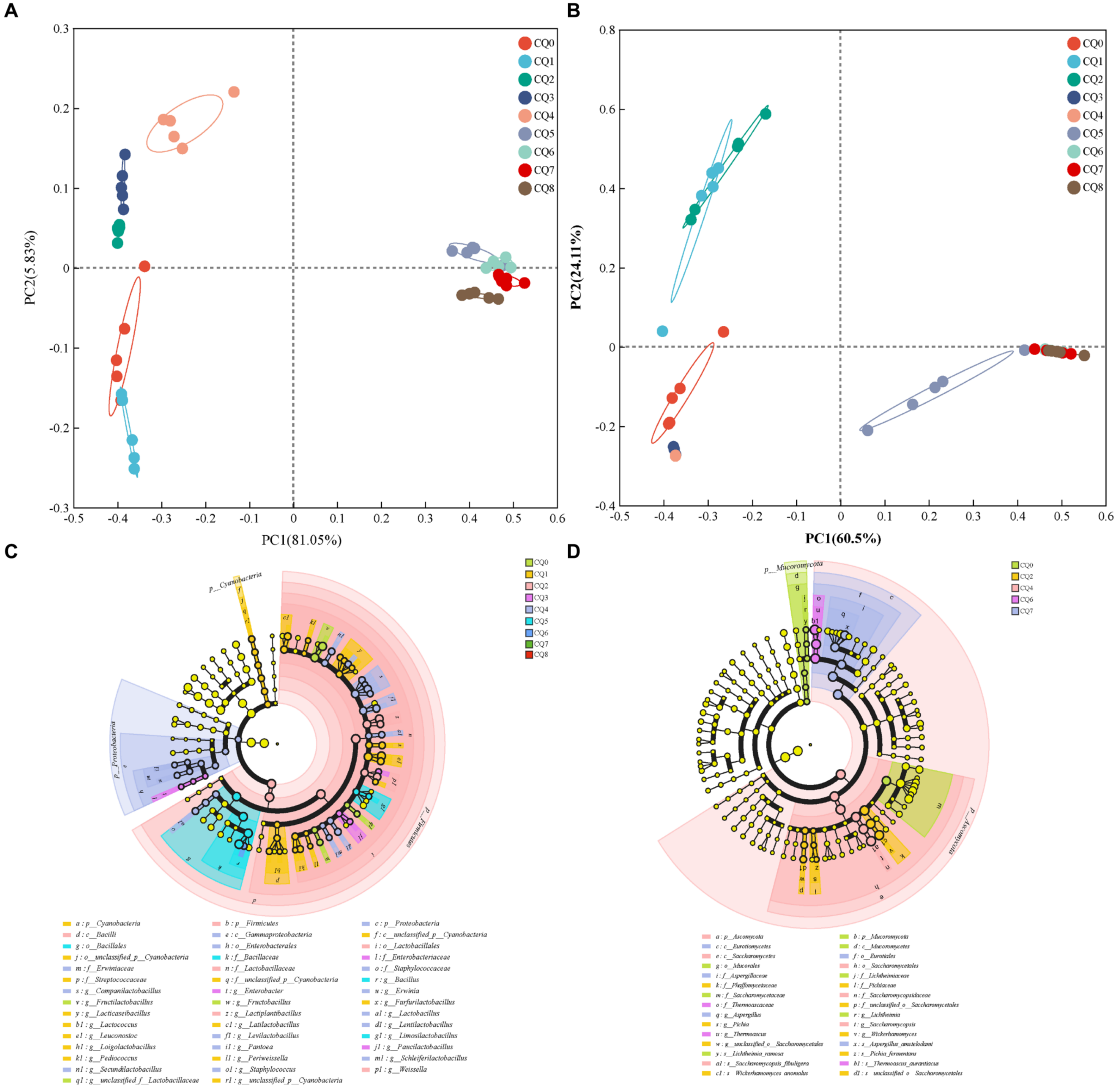
Principal component analysis of bacterial **(A)** and fungi **(B)** community composition of Qupi during the forming FHD from Qupi; LEfSe-based marker analysis of Qupi bacteria **(C)** and fungi **(D)**.

Figures 5 show the species composition at the genus and species levels, respectively, for the nine points of Qupi. By analyzing the microbial community structure through principal components, it can be observed that PC1 and PC2 explained 86.88% of the variance in the bacterial community (Figure 6A) and a total of 84.61% in the fungi community (Figure 6B). The results showed that there were large variations in microbial community changes at different stages, especially from CQ5 to CQ8, compared to the previous five stages, indicating that the substantial temperature changes were the main factor influencing the changes in microbial community structure.

As for the structural composition of the bacterial community, a total of 84 bacterial OTUs were identified from nine points of Qupi during the forming FHD from Qupi and clustered into 5 phyla, 45 genera, and 71 species. One shared dominant bacterial phylum *Firmicutes* was detected based on the phylum classification level. During the warming period, CQ0 included seven dominant bacterial genera, such as *Fructilactobacillus* (40.23%), *Leuconostoc* (29.46%), and *Lactiplantibacillus* (17.36%). Additionally, *Fructilactobacillus sanfranciscensis* (40.20%), *Leuconostoc citreum* (28.83%), and *Lactiplantibacillus plantarum* (15.44%) accounted for 80% of the bacterial community. CQ1 included seven dominant bacterial genera, such as *Leuconostoc* (37.93%), *Lactiplantibacillus* (22.90%), and *Fructilactobacillus* (14.10%). *L. plantarum* (21.01%), *L. citreum* (35.50%), and *F. sanfranciscensis* (14.08%), and other five bacterial community with 80% abundance. CQ2 included seven dominant bacterial genera, such as *Fructilactobacillus* (36.70%), *Lactiplantibacillus* (26.63%), and *Leuconostoc* (12.22%). *F. sanfranciscensis* (36.70%), *L. plantarum* (24.09%), and *L. citreum* (11.54%) and four other bacterial flora with 80% abundance. CQ3 included seven dominant genera, such as *Fructilactobacillus* (31.83%), *Lactiplantibacillus* (23.29%), and *Leuconostoc* (12.73%). The abundance of five bacterial community, such as *F. sanfranciscensis* (31.83%), *L. plantarum* (22.16%), and *L. citreum* (12.21%) accounted for 80% of the total. During the high-temperature period, CQ4 included seven dominant bacterial genera, such as *Lactiplantibacillus* (20.38%), *Fructilactobacillus* (14.10%), and *Leuconostoc* (12.79%). *F. sanfranciscensis* (20.09%), *L. plantarum* (19.48%), and *L. citreum* (12.33%) and seven bacterial community with 80% abundance. CQ5 included eight dominant bacterial genera, such as *Bacillus* (64.92%), *Saccharopolyspora* (10.98%), and *Fructilactobacillus* (4.65%). *Bacillus licheniformis* (63.94%), *S. rectivirgula* (10.04%), and *F. sanfranciscensis* (4.65%), and other five bacterial community with 80% abundance. At maturity, CQ6 included eight dominant bacterial genera, such as *Bacillus* (59.39%), *Saccharopolyspora* (21.42%), and *Fructilactobacillus* (3.12%). *B. licheniformis* (57.81%), *S. rectivirgula* (21.42%), and *F. sanfranciscensis* (3.12%), and five bacterial flora with 80% abundance. CQ7 included seven dominant bacterial genera, such as *Bacillus* (61.07%), *Saccharopolyspora* (23.08%), and *Fructilactobacillus* (2.86%). *B. licheniformis* (60.15%), *S. rectivirgula* (23.08%), and *F. sanfranciscensis* (3.12%), and other five bacterial community with 80% abundance. When proceeding to CQ8, the Qupi reached a mature state with eight dominant bacterial genera, including *Bacillus* (43.91%), *Saccharopolyspora* (35.06%), and *Fructilactobacillus* (6.03%), and *B. licheniformis* (43.25%), *S. rectivirgula* (35.05%), and *F. sanfranciscensis* (6.03%), and five bacterial community with 80% abundance.

A total of 97 fungi OTUs were detected from 9 points of Qupi during the forming FHD from Qupi and clustered into 4 phyla, 58 genera, and 82 species. One dominant fungi phylum *Ascomycota,* was detected based on the phylum classification level. During the warming period, CQ0 included three dominant fungi genera *Saccharomycopsis* (65.87%), *Kazachstania* (29.49%), and *Alternaria* (1.03%). *Saccharomycopsis fibuligera* (65.87%), *Kazachstania humilis* (13.86%), and *Kazachstania exigua* (12.48%), and three species of fungi community with 80% abundance. CQ1 included *Saccharomycopsis* (33.28%), *Wickerhamomyces* (29.49%), and *Kazachstania* (17.71%), four dominant fungi genera. *Wickerhamomyces anomalus* (43.10%), *S. fibuligera* (33.28%), and *K. exigua* (13.90%), and three species of fungi community with 80% abundance. CQ2 included three dominant fungi genera *Saccharomycopsis* (69.00%), *Wickerhamomyces* (26.19%), and *Kazachstania* (3.24%). *W. anomalus* (69.00%) and *S. fibuligera* (26.19%) with 80% abundance. CQ3 included two dominant fungi genera, *Saccharomycopsis* (98.51%) and *Wickerhamomyces* (1.10%). *S. fibuligera* (98.51%) and *W. anomalus* (1.10%) were predominant. During the high-temperature period, CQ4 included one dominant fungi genus, *Saccharomycopsis* (99.89%), which was dominated by *S. fibuligera* (99.89%). CQ5 consisted of 2 dominant fungi genera, *Thermoascus* (63.53%) and *Saccharomycopsis* (36.15%). The dominant fungi genera were dominated by *T. aurantiacus* (63.53%) and *S. fibuligera* (36.15%). At maturity, CQ6 included two dominant fungi genera, *Thermoascus* (90.26%) and *Saccharomycopsis* (9.25%), dominated by *T. aurantiacus* (90.26%) and *S. fibuligera* (9.25%). CQ7 included four dominant fungi genera, including *Thermoascus* (62.52%), *Aspergillus* (28.55%), and *Saccharomycopsis* (7.68%). *T. aurantiacus* (62.52%) and *A. amstelodami* (24.25%) with 80% abundance. CQ8 had 3 dominant fungi genera, including *Thermoascus* (76.51%), *Aspergillus* (13.81%), and *Saccharomycopsis* (8.88%). *T. aurantiacus* (76.51%), and *A. amstelodami* (10.81%) with 80% abundance.

#### 3.4.3 Changes in microbial community succession

Bacteria common to the nine points of Qupi during the forming FHD from Qupi included *F. sanfranciscensis*, *Paucilactobacillus vaccinostercus*, *Lactiplantibacillus mudanjiangensis*, *Lacticaseibacillus paracasei*, *Weissella confusa*, and *Latilactobacillus sakei*. The main dominant bacteria (relative abundance more than 10%) detected during the warming period were *F. sanfranciscensis*, *L. citreum*, and *L. plantarum*, with an average relative abundance of 30.70, 22.02, and 20.83%, respectively. The bacterial community was greatly affected by the high temperature during the warming period due to increased temperature. As such, the community differences between the 2 stages, CQ4 and CQ5, were large. In addition to the 7 dominant bacteria in the warming period, the relative abundance of *B. licheniformis* increased from less than 0.01 to 9.34% in the CQ4 stage. In CQ5, the relative abundance of *B. licheniformis* increased to 63.94%, which was the highest. The relative abundance of *S. rectivirgula* increased from less than 0.01 to 10.98%. The relative abundance of *unclassified_c_Actinomycetia* increased from less than 0.01 to 1.87%. Additionally, the relative abundance of three dominant bacteria, including *W. confusa*, decreased to a lower level, and the relative abundance of *L. sakei* was below 0.01%. During the maturation period, the changes in the composition and abundance of the dominant bacterial community were more different from the CQ0 to CQ4 stages compared to the CQ5 stage. The main bacterial communities were dominated by *B. licheniformis* and *S. rectivirgula*, with an average relative abundance of 53.74 and 26.52%, respectively. The abundance of *S. rectivirgula* and *unclassified_c__Actinomycetia* increased to the highest in CQ8, with a relative abundance of 35.05 and 4.14%, respectively. In conclusion, the predominant bacterial community in the Qupi at nine points during FHD preparation evolved from *F. sanfranciscensis*, *L. citreum,* and *L. plantarum* to *B. licheniformis* and *S. rectivirgula*, which constitute the core bacterial community of FHD.

The common fungus in the nine points of Qupi during the forming FHD from Qupi was *S. fibuligera* and *W. anomalus*. The species and distribution levels of their dominant fungi varied greatly between the warming and high-temperature periods. The main dominant fungi (relative abundance of more than 10%) in the CQ0 stage were *S. fibuligera*, *K. exigua*, and *K. humilis*. In the CQ1, CQ2, and CQ3 stages, the dominant fungi evolved into *S. fibuligera, W. anomalus,* and *K. exigua*, with an average relative abundance of *K. humilis* dropping to lower levels. At the CQ4 stage, *S. fibuligera* (99.89%) was the absolutely dominant fungus. At the CQ5 stage, the relative abundance of *S. fibuligera* decreased to 36.15%, while *T. aurantiacus* increased from less than 0.01 to 63.53%. At the maturation period, *T. aurantiacus* dominated with a relative abundance of 90.26, 62.52, and 76.51% at the CQ6 to CQ8 stages, respectively. In addition, the relative abundance of *S. fibuligera* was reduced to less than 10%, and *A. amstelodami* was the dominant fungus in CQ7 and CQ8, with a relative abundance of 24.59 and 10.81%, respectively. In conclusion, the main fungi community in the Qupi at nine points during FHD preparation evolved from *S. fibuligera*, *K. exigua,* and *K. humilis* to *T. aurantiacus*, *A. amstelodami,* and *S. fibuligera*, which constitute the core fungi community of FHD.

#### 3.4.4 Biomarker analysis

The microbial community composition of Qupi samples at nine points during the forming FHD from Qupi was characterized using LEfSe multilevel species difference discriminant analysis. Different color nodes indicate microbial taxa that were significantly enriched in the corresponding groups and had a significant effect on the intergroup differences; yellowish nodes indicate microbial taxa that did not have a significant effect on the intergroup differences.

As for bacterial composition, at the species level, 40 significantly different species were detected during the forming FHD from Qupi (Figure 6C, LDA > 3, *p* < 0.05). A total of 3, 15, 3, 3, 13, 2, and 1 significantly different species were detected at the CQ0 to CQ6 stages, respectively. Among them, *F. sanfranciscensis*, *L. citreum*, *L. plantarum*, *unclassified_g_Enterobacter*, *Companilactobacillus mindensis, B. licheniformis*, and *Bacillus safensis* were used as important markers for each stage from CQ0 to CQ6, respectively. No significantly different species were detected at the CQ7 and CQ8 stages.

As for fungi composition, at the species level, seven significantly different species were detected during the forming FHD from Qupi, namely *Lichtheimia ramosa*, *W. anomalus*, *Pichia fermentans, S. fibuligera*, *T. aurantiacus*, *A. amstelodami*, and *unclassified__o_Saccharomycetales* (Figure 6D, LDA > 2, *p* < 0.05). Among them, *L. ramosa*, *W. anomalus*, *S. fibuligera*, *T. aurantiacus,* and *A. amstelodami* were used as important markers for the CQ0, CQ2, CQ4, CQ6, and CQ7 stages, respectively. Significantly different species were not detected at the CQ1, CQ3, CQ5, and CQ8 stages.

### 3.5 Dynamics of volatile substances

#### 3.5.1 Volatile substance composition and distribution

**Figure 7:**
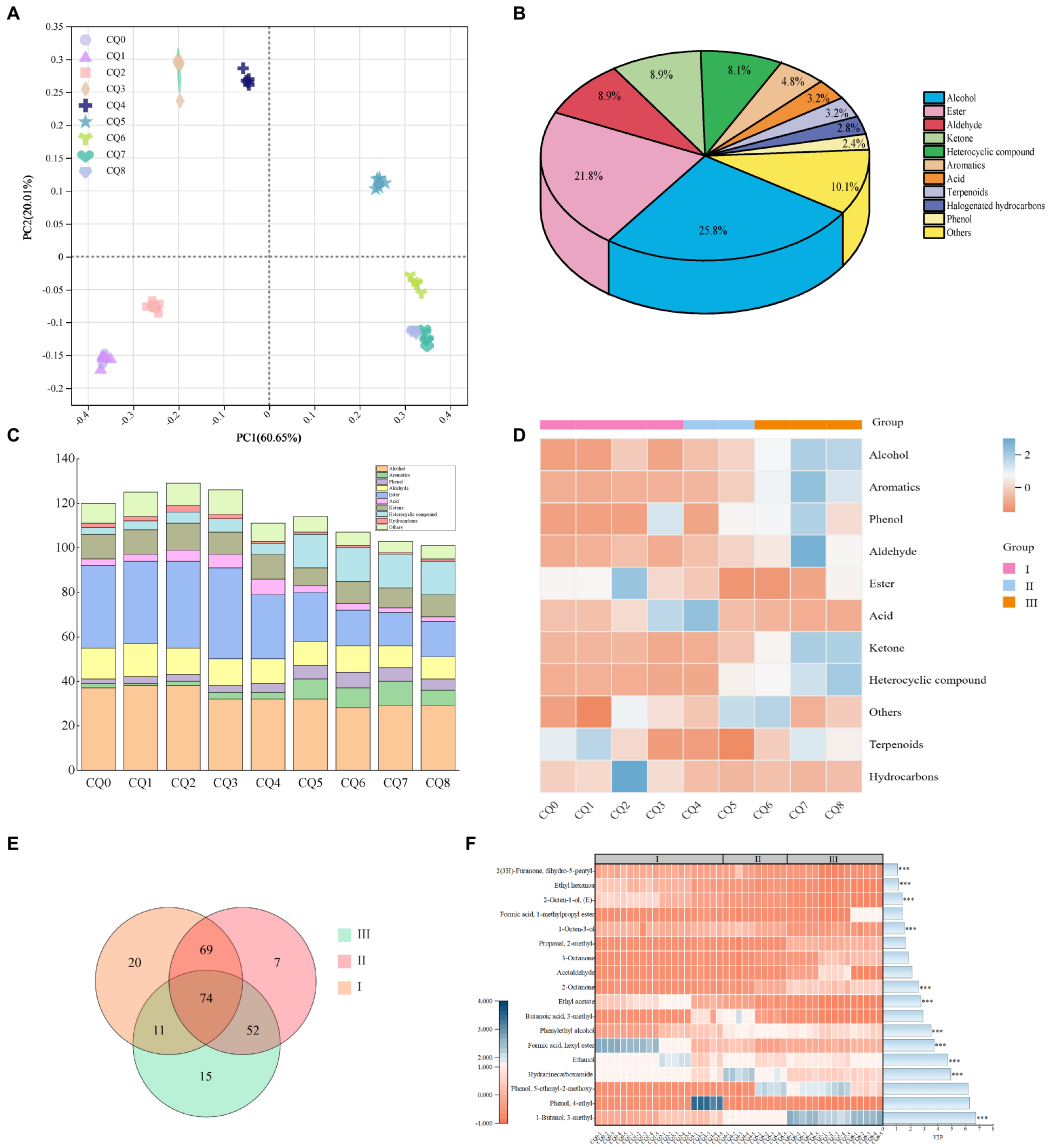
Graphs representing the PCoA analysis of volatile substances in Qupi during the forming FHD from Qupi **(A)**, volatile substance classification **(B)**, distribution of volatile substance types and relative contents **(C,D)**, and distribution of important volatile substances in the Venn diagrams and thermograms **(E,F)**.

A total of 248 volatiles were detected using HS-SPME-GC-MS to detect the volatiles in Qupi at nine points during the forming FHD from Qupi. The variability of FHD volatiles was analyzed by principal coordinate analysis (PCA), and the variations of volatiles were mainly concentrated in the first principal component axis (PC1) and the second principal component axis (PC2), which were 60.65 and 20.01%, respectively, accounting for 80.66% of the total variation (Figure 7A). The distribution of scatter positions within the group at different stages of FHD preparation was not centralized, indicating differences in the type of volatile metabolites. The samples were distributed very close to each other at stages CQ0, CQ1, and CQ2, and closer between stages CQ6–CQ8, with overlapping distributions at stages CQ7 and CQ8, suggesting that the volatiles at the maturation period were stable and reached a mature and stable state.

Figure 7B shows the highest average relative content of alcohols (25.8%), followed by esters (21.8%), aldehydes (8.9%), and ketones (8.9%) in the samples. Among them, 33 volatiles accounted for about 80% of the total content, mainly including alcohols, esters, aldehydes, and ketones. During the forming FHD from Qupi, the amount of alcohols and aldehydes showed a little change, the amount of aromatic and heterocyclic compound volatiles gradually increased, and the types of esters gradually decreased (Figure 7C). The number of volatile substances reached the highest during the warming period, with a total of 174 volatile substances detected. The total content of volatile substances showed an increasing trend, with the CQ2 and CQ3 stages having the most abundant types of volatile substances. The warming period included 50 esters, 46 alcohols, 22 ketones, 20 heterocyclic compounds, etc. The high-temperature period included a total of 154 volatile substances. In the high-temperature period, a total of 154 volatiles were detected, including 32 esters, 42 alcohols, 12 aldehydes, and 12 ketones. The high temperature inhibited the growth and metabolism of some microorganisms, so the number of esters and terpenes decreased, but the number of aromatic compounds and heterocyclic compounds increased. At maturity, a total of 126 volatiles were detected, including 19 esters, 35 alcohols, 12 aldehydes, 11 ketones, and 11 aromatic compounds.

As shown in Figure 7D, the content of acids and halogenated hydrocarbons showed an overall decreasing trend during the forming FHD from Qupi. The contents of alcohols, aldehydes, phenols, ketones, aromatic compounds, and heterocyclic compounds showed a gradual increase throughout the preparation cycle. The content of hydrocarbons and terpenes did not change significantly. The average content of alcohols increased from 464.65 μg/kg to 955.59 μg/kg, with a slow increase during the warming period and a faster increase during the high temperature and maturation periods. Aldehydes increased from 157.52 μg/kg to 370.39 μg/kg, phenols from 90.94 μg/kg to 292.67 μg/kg, ketones from 30.42 μg/kg to 252.61 μg/kg, heterocyclic compounds from 9.44 μg/kg to 84.55 μg/kg, and aromatic compounds from 5.88 μg/kg to 70 μg/kg. The aromatic compounds increased from 5.88 μg/kg to 70.81 μg/kg. The esters and acids showed a tendency to decrease and then increase, with esters decreasing from 432.43 μg/kg to 8.50 μg/kg and acids decreasing from 59.99 μg/kg to 178.37 μg/kg and then increasing to 215.80 μg/kg. Acids showed the smallest change in content during the process.

The number of shared volatiles in the three main periods of the forming FHD from Qupi, namely, the warming, high temperature, and maturation periods, was visualized using Venn diagrams. The critical differential volatiles were screened by PLS-DA predictor variable significance VIP > 1 and *t*-test (*p* < 0.05), and the clustered heatmaps and VIP bar graphs showed the changes in the significance and relative contents of differential volatiles at different periods (Figures 7E,F). A total of 74 shared volatiles and 20, 7, and 15 unique volatiles were detected. Based on the VI*p* values, 1-butanol-3-methyl-, hydrazinecarboxamide, ethanol, phenylethyl alcohol, ethyl acetate, 2-octanone, 1-octen-3-ol, formic acid-hexyl ester, (E)-2-octen-1-ol, ethyl hexanoate, and 2(3H)-furanone-dihydro-5-pentyl-a total of 11 volatiles had a significant effect on the flavor of the Qupi, with alcohols having the highest VIP values, namely 1-butanol-3-methyl-. The content of 1-butanol-3-methyl- increased from 18.07 μg/kg (CQ0) to 395.85 μg/kg (CQ8), The content of alcohol increased from 98.26 μg/kg (CQ0) to 227.49 μg/kg (CQ8), while the content of phenylethyl alcohol increased from 13.18 μg/kg (CQ0) to 17.5 μg/kg (CQ8). (E)-2-octen-1-ol, content decreased from 55.21 μg/kg (CQ0) to 9.59 μg/kg (CQ8). Hydrazinecarboxamide increased from 106.22 μg/kg (CQ0) to 162.53 μg/kg (CQ8). 2-Octanone was also an important flavor substance, and its content increased from 1.42 μg/kg (CQ0) to 148.41 μg/kg (CQ8). These substances may be the key substances in determining the flavor of Fengxiangxing baijiu. Additionally, some volatiles with no significant differences in the expression were detected. Among them, 1-octen-3-ol with a strong mushroom odor, was the highest in the high-temperature period (47.589 μg/kg). Geosmin, an earthy substance in the Qupi, was also produced in the high-temperature period, which reached its highest level at the maturation of Qupi. These substances are one of the sources of off-flavors in Qupi (Zheng et al., 2018; Deng et al., 2023).

#### 3.5.2 Metabolic pathway enrichment analysis

**Figure 8:**
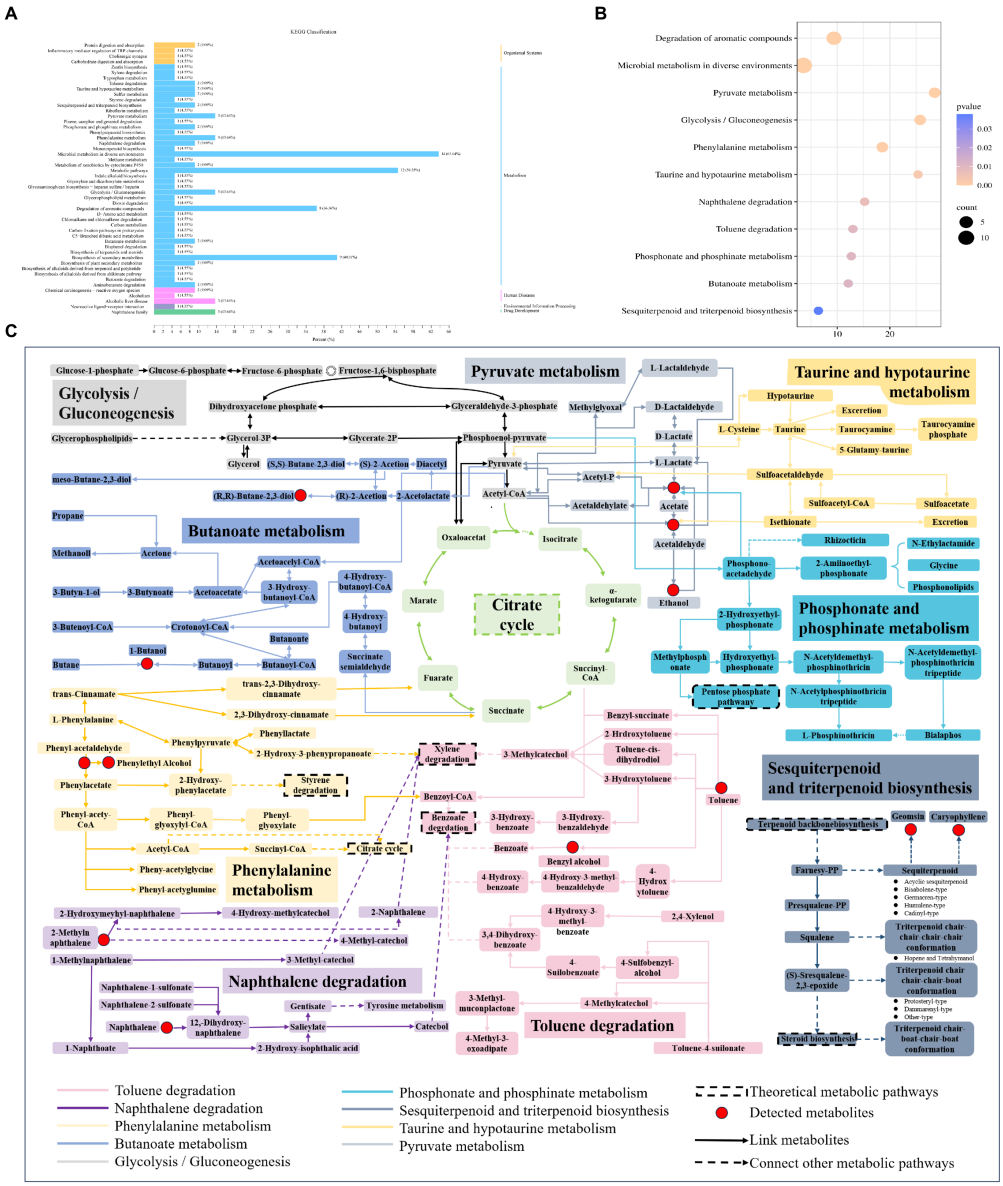
KEGG pathway classification **(A)**, analysis of the 11 key metabolic pathways **(B)** and related flavour formation pathways based on key metabolic pathways **(C)** during the forming FHD from Qupi.

The nine points of differential volatiles were analyzed by pathway enrichment using the KEGG database, which involved 51 metabolic pathways, and the number of volatiles annotated in each KEGG pathway and the metabolic pathways of the differential volatiles are shown in Figure 8A, microbial metabolism in diverse environments was the most important pathway annotated in all KEGG pathways, with the highest number of volatiles annotated, which were closely related to the synthesis and metabolism of flavor substances in the samples. This was followed by metabolic pathways, biosynthesis of secondary volatiles, and the degradation of aromatic compounds. The 11 key metabolic pathways (*p* < 0.05) included the degradation of aromatic compounds, microbial metabolism in diverse environments, pyruvate metabolism, glycolysis/gluconeogenesis, phenylalanine metabolism, taurine and hypotaurine metabolism, naphthalene degradation, toluene degradation, phosphonate and phosphinate metabolism, butanoate metabolism, and sesquiterpenoid and triterpenoid biosynthesis (Figure 8B). The key metabolic pathways of microorganisms during the forming FHD from Qupi were summarized based on the metabolic pathways, detected volatiles, and KEGG pathways (Figure 8C).

### 3.6 Correlation analysis

**Figure 9:**
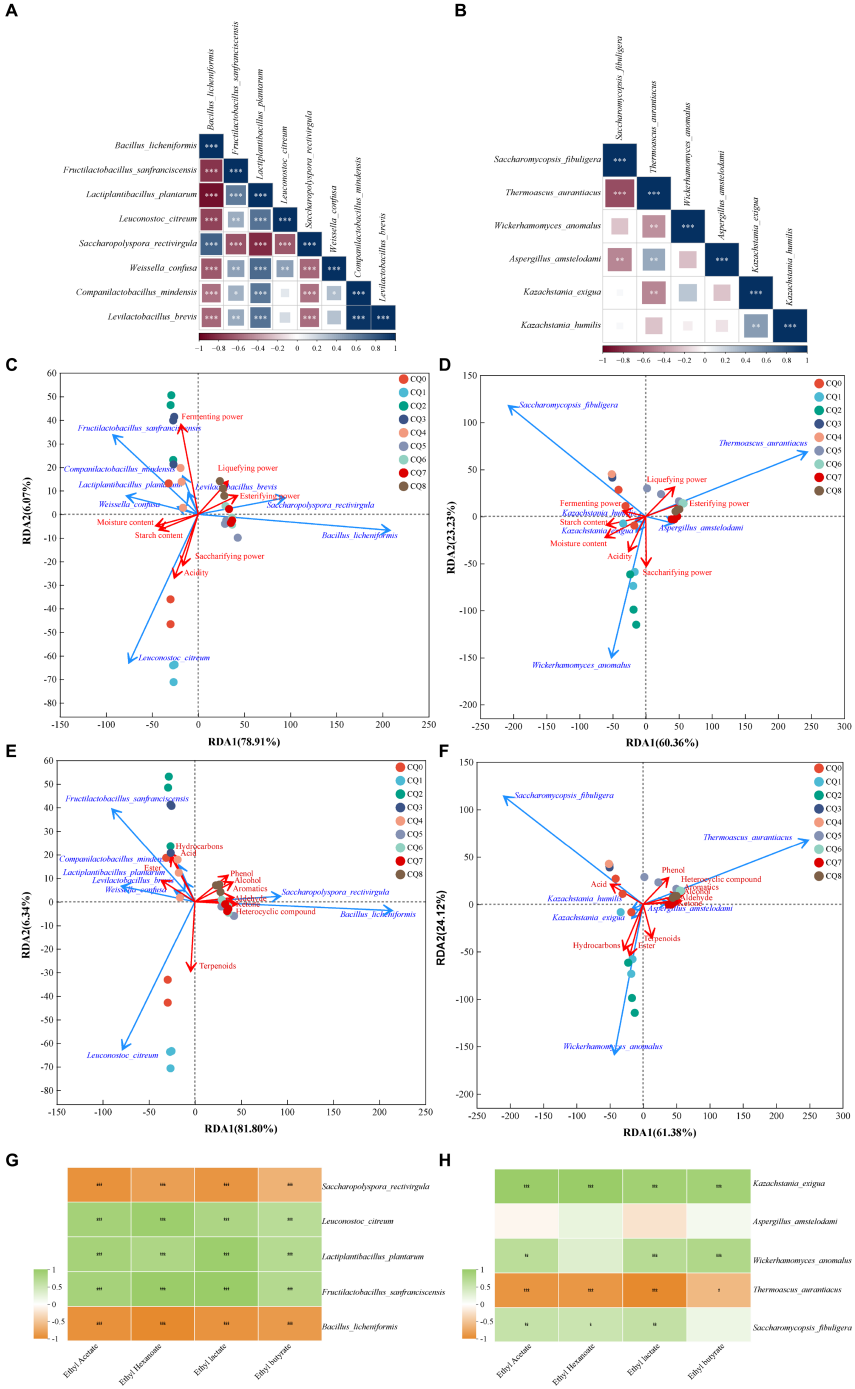
Correlation analysis of Qupi microorganisms during the forming FHD from Qupi (**A**: bacteria, **B**: fungi); correlation analysis of dominant bacteria and fungi with fermentation characteristics, respectively (**C**: bacteria, **D**: fungi); correlation analysis of dominant bacteria and fungi with volatiles (**E**: bacteria, **F**: fungi); correlation analysis of four esters with dominant bacteria and fungi (**G**: bacteria, **H**: fungi).

The dominant microorganisms with an average relative abundance greater than 1% in the Qupi during the forming FHD from Qupi were analyzed using Spearman correlation analysis, and the results are shown in Figure 9. As shown in Figure 9A, *B. licheniformis* showed a significant positive correlation with *S. rectivirgula*, and a negative correlation with the rest of the six bacterial species. On the contrary, *L. plantarum* showed a positive correlation with these six dominant bacteria. *B. licheniformis*, *F. sanfranciscensis*, *L. plantarum*, and *S. rectivirgula* showed a positive correlation. *L. plantarum* and *S. rectivirgula* showed a significant correlation with each other. As shown in Figure 9B, *T. aurantiacus* showed a significant positive correlation with *W. anomalus*, *K. exigua,* and *K. humilis*. *T. aurantiacus* showed a significant negative correlation with *K. exigua*, while *K. humilis* showed a significant negative correlation with *K. exigua*, while *K. humilis* showed no correlation and showed a positive correlation with the remaining three dominant fungi. It is evident that these dominant bacterial and fungi taxa play a major role in dominant microbial interactions.

The correlation between the eight dominant bacteria and fermentation characteristics during the forming FHD from Qupi was analyzed using RDA. The cumulative explanation of RDA1 and RDA2 was 87.93% (Figure 9C). Among them, the arrow of fermenting power was longer and was most affected by the abundance of the dominant bacterial community. *B. licheniformis* and *S. rectivirgula* were negatively correlated with moisture, acidity, starch content, fermenting power, and saccharifying power, respectively, and positively correlated with the esterifying power and liquefying power. Unlike *F. sanfranciscensis, L. plantarum, L. citreum,* and *W. confusa,* the opposite was true. *C. mindensis* and *Levilactobacillus brevis* were positively correlated with moisture, acidity, starch content, and fermenting power, and negatively correlated with saccharifying power, esterifying power, and liquefying power, respectively. Based on the order of importance explained by the environmental factor variables, it can be inferred that moisture was the most important driver of the change in microbial community abundance during the forming FHD from Qupi, followed by esterifying power, starch content, fermenting power, acidity, liquefying power, and saccharifying power (Table 2A).

**Table 2:** Importance ranking and significance test results.

Environmental factor	RDA1	RDA2	*r*^2^	*p* value
**A: dominant bacteria and fermentation characteristics**				
Moisture content	−0.9971	−0.0759	0.9137	0.001
Acidity	−0.7397	−0.673	0.5791	0.001
Starch content	−0.9928	−0.1194	0.7791	0.001
Esterifying power	0.9895	0.1446	0.8015	0.001
Liquefying power	0.9398	0.3418	0.5187	0.001
**B: dominant fungi and fermentation characteristics**				
Moisture content	−0.935	−0.3546	0.8853	0.001
Acidity	−0.6158	−0.7879	0.4053	0.005
Starch content	−0.9821	−0.1882	0.7316	0.001
Esterifying power	0.9618	0.2739	0.8307	0.001
Liquefying power	0.8262	0.5634	0.5883	0.001
**C: dominant bacteria and volatiles**				
Alcohol	0.9834	0.1816	0.677	0.001
Aromatics	0.9988	0.0482	0.6712	0.001
Phenol	0.9636	0.2673	0.5382	0.001
Aldehyde	0.9998	0.021	0.4954	0.003
Ester	−0.9804	0.1972	0.5235	0.002
Acid	−0.848	0.5301	0.3805	0.003
Ketone	1	0.0045	0.583	0.002
Heterocyclic compound	1	−0.0062	0.7938	0.001
Terpenoids	−0.1918	−0.9814	0.3112	0.012
Hydrocarbons	−0.8292	0.5589	0.417	0.006
**D: dominant fungi and volatiles**				
Alcohol	0.9964	0.0842	0.6768	0.001
Aromatics	0.9787	0.2052	0.6855	0.001
Phenol	0.8168	0.5769	0.4794	0.004
Aldehyde	0.9969	0.0784	0.4698	0.001
Ester	−0.5567	−0.8307	0.6963	0.001
Acid	−0.9372	0.3488	0.5824	0.001
Ketone	0.994	0.1092	0.6771	0.001
Heterocyclic compound	0.979	0.2039	0.8414	0.001
Terpenoids	0.3479	−0.9375	0.2744	0.021
Hydrocarbons	−0.3943	−0.919	0.7296	0.001

The correlation between the six dominant fungi and fermentation characteristics during the forming FHD from Qupi is shown in Figure 9D. The cumulative explanation of RDA1 and RDA2 was 84.23%. Among them, moisture, starch content, fermenting power, liquefying power, and esterifying power showed longer arrows and were most affected by the abundance of the dominant fungi community. *S. fibuligera* was negatively correlated with the esterifying power, liquefying power, and saccharifying power and positively correlated with acidity, moisture, starch content, and fermenting power. However, *T. aurantiacus* showed the opposite trend. *W. anomalus* was negatively correlated with the esterifying power, liquefying power, and fermenting power and positively correlated with acidity, moisture, starch content, and saccharifying power. *K. exigua* and *K. humilis* were negatively correlated with the esterifying power and liquefying power and positively correlated with acidity, moisture, starch content, and saccharifying power, respectively. *K. exigua* and *K. humilis* were negatively correlated with esterifying power and liquefying power, respectively, and positively correlated with acidity, moisture, starch content, saccharifying power, and fermenting power. *A. amstelodami* was negatively correlated with moisture, starch content, and fermenting power and positively correlated with acidity, esterifying power, liquefying power, and saccharifying power. Based on the order of importance, it can be seen that moisture was the most important driver of the change in fungi colony abundance during the forming FHD from Qupi, followed by esterifying power, starch content, liquefying power, saccharifying power, acidity, and fermenting power (Table 2B).

The correlation between the eight dominant bacteria and volatile substances during the forming FHD from Qupi is shown in Figure 9E. The cumulative explanation of RDA1 and RDA2 was 88.14%. Among them, *B. licheniformis* and *S. rectivirgula* were positively correlated with six substances, such as alcohols, aromatic compounds, and phenols, and negatively correlated with four substances, such as esters, acids, and hydrocarbons, respectively. *F. sanfranciscensis, L. plantarum, C. mindensis,* and *L. brevis* were positively correlated with three substances, namely esters, acids, and hydrocarbons, and negatively correlated with the remaining seven substances. *W. confusa* and *L. citreum* were positively correlated with 4 substances of esters, acids, terpenes, and hydrocarbons, respectively, and negatively correlated with the remaining 6 substances. Based on the order of importance, it can be inferred that the content of heterocyclic compounds was most affected by the change in the abundance of microbial community during the forming FHD from Qupi, followed by alcohols, aromatic compounds, ketones, phenols, esters, aldehydes, hydrocarbons, acids, and terpenoids (Table 2C).

The correlation between the six dominant fungi and volatile substances during the forming FHD from Qupi is shown in Figure 9F. The cumulative explanation of RDA1 and RDA2 was 85.50%. Among them, *S. fibuligera* was negatively correlated with seven substances, such as alcohols, aromatic compounds, phenols, and aldehydes, and positively correlated with acids, esters, and hydrocarbons. *T. aurantiacus* was opposite to *S. fibuligera*. *W. anomalus, K. exigua*, and *K. humilis* were negatively correlated with six substances, including alcohols, aromatic compounds, phenols, and aldehydes, and positively correlated with four substances, namely acids, esters, terpenes, and hydrocarbons. *A. amstelodami was* negatively correlated with six substances, including alcohols, aromatic compounds, phenols, and aldehydes, and positively correlated with four substances, namely acids, esters, terpenes, and hydrocarbons. *A. amstelodami* was negatively correlated with three substances, including phenols, terpenes, and hydrocarbons, and positively correlated with the remaining seven substances. According to the order of importance, it can be inferred that the heterocyclic compounds were most affected by the changes in the abundance of fungi colonies during the forming FHD from Qupi, followed by hydrocarbons, esters, aromatic compounds, ketones, alcohols, acids, phenols, aldehydes, and terpenoids (Table 2D). In conclusion, the results of the correlation analysis indicated that the dominant microbial community had a strong influence and close relationship with the fermentation characteristics and volatiles.

In Fengxiangxing baijiu, ethyl acetate, ethyl hexanoate, ethyl lactate, and ethyl butyrate are the main volatile metabolites involved in the formation of Fengxiangxing baijiu style, with ethyl acetate being the most abundant ester (Liu et al., 2022). The correlation of the dominant bacteria, *F. sanfranciscensis*, *L. plantarum*, *L. citreum*, *B. licheniformis*, and *S. rectivirgula,* and the dominant fungi, *S. fibuligera*, *W. anomalus*, *K. exigua*, *T. aurantiacus*, and *A. amstelodami,* with ethyl acetate, ethyl hexanoate, ethyl lactate, and ethyl butyrate in Fengxiangxing baijiu were analyzed. As shown in Figure 9G, *F. sanfranciscensis*, *L. plantarum,* and *L. citreum* were significantly positively correlated with the content of ethyl acetate, ethyl hexanoate, ethyl lactate, and ethyl butyrate. *B. licheniformis* and *S. rectivirgula* were significantly negatively correlated with the content of these four esters. Figure 9H shows that *S. fibuligera*, *W. anomalus,* and *K. exigua* were significantly positively correlated with the content of these four esters. The abundance of *T. aurantiacus* was significantly negatively correlated with the content of these four esters. Only *A. amstelodami* showed the lowest correlation with the content of these four esters.

## 4 Discussion

In this study, the dynamic changes in the microbial diversity and their abundance at nine points of the forming FHD from Qupi were analyzed using the PacBio sequencing technology. The moisture and temperature during the forming FHD from Qupi strongly influenced the fermentation characteristics, microbial composition and relative abundance levels, and volatiles at different stages of fermentation, thereby prompting the synergistic, adaptive, and stabilizing microbial communities to regulate Qupi maturation and promote the conversion of beneficial substances to form a stable microsystem for Qupi process.

The fermentation characteristics of Qupi are important indicators reflecting the quality and maturity of Daqu and have a certain connection with the microbial composition of Qupi. Jiangxiangxing Daqu due to the process of preparing the Qupi is at a high temperature for a long time, the number of yeast is low, the esterifying power, fermenting power, saccharifying power and liquefying power are low, and the acidity value of the mature Jiangxiangxing Daqu is high (Yi et al., 2019; Zhu et al., 2022; Deng et al., 2023). Qingxiangxing Daqu has higher esterifying power, saccharifying power, fermenting power, and liquefying power due to the lower preparation temperature, higher number of microbial species, and lower acidity value (Zheng et al., 2014; Deng et al., 2023). FHD had high esterification, saccharification, and fermenting power but low liquefying power. Therefore, it can be inferred that the fermentation characteristics of different aroma types of Daqu are unique, and the peak temperature of Daqu preparation has a significant influence on the fermentation characteristics.

During the forming FHD from Qupi, the microbial community, especially the filamentous microorganisms, exert different species, morphology, abundance, distribution, nutrient growth, reproductive development stages, and volatiles, which play a decisive role in the quality formation of Daqu. During the warming period, the microorganisms began to multiply and grow on the surface and then gradually spread to the interior of the Qupi through volatilization and utilization of moisture. *F. sanfranciscensis*, *L. citreum*, *L. plantarum, S. fibuligera*, *K. exigua*, and *K. humilis* dominated the initial community structure formation, and their abundance showed a slight decrease at the CQ0-CQ3 stages. Although the specific roles of *Lactiplantibacillus* and *Fructilactobacillus* in the process of baijiu brewing are less frequently reported, they showed a dominant role in this study. The abundance of *Fructilactobacillus* was significantly correlated with the acid-producing capacity of the samples (r = 0.776, *p* < 0.001). Therefore, it was hypothesized to be the main microorganism responsible for the decreased acidity in Qupi. During the high-temperature period, the temperature of the Qupi continued to rise, the consumption of moisture increased, and the microbial growth and reproduction on the surface of Qupi decreased. Meanwhile, the microorganisms shifted to the interior of the Qupi to reproduce and grow, and the diversity and the complexity of the fungi community gradually decreased. As a result, FHD was susceptible to the differences in temperature, humidity, and oxygen during the preparation process, and *S. fibuligera, B. licheniformis*, and *T. aurantiacus* became the dominant community. *S. fibuligera* has well-developed microbial characteristics and pseudohyphae, which reached the highest during the high-temperature period. The present study results proved that the white pseudohyphae of *S. fibuligera* were responsible for the white color on the surface of Qupi. Subsequently, during the CQ5 stage, heat-resistant and thermophilic microorganisms, such as *B. licheniformis and T. aurantiacus*, multiplied rapidly to prepare for the maturation of Qupi. Moisture was the main driver of microbial community changes in FHD. During the maturation period, the temperature of Qupi slowly decreased, the moisture decreased, the microbial survival conditions became worse, some microorganisms gradually started to form spores and sporocarps, and the Qupi entered the maturation period. *B. licheniformis*, *S. rectivirgula, T. aurantiacus,* and *A. amstelodami* became the main microflora during the maturation period. Among them, the main dominant fungus *A. amstelodami* was closely related to the sensory formation of FHD, with certain drought and heat resistance (Shen, 2007), which mainly increases through sexual reproduction in the form of producing yellow ascospores and is easy to form under higher temperatures and lower moisture environments, allowing the proliferation inside the quebracho and during the maturation period. It is reported that the bright yellow closed capsules produced by *A. amstelodami* are the cause of the formation of the “dense golden-yellow spots inside,” a typical characteristic of FHD. Moreover, the relative abundance of *Aspergillus* is significantly higher in the premium grade than in the first grade, suggesting that *Aspergillus* plays an important role in the quality formation of Daqu (Xiang et al., 2022). The grayish-white color of the FHD interior is associated with the grayish-white mycelia of *T. aurantiacus*. *T. aurantiacus* is a dominant fungus in many liquor brewing tunes, causing differences in the quality of large tunes. *T. aurantiacus* has an optimal growth and reproduction temperature of 45°C-48°C and produces enzymes with high thermal stability. Therefore, it can grow and reproduce stably in a high heat environment (Cai et al., 2021). Additionally, *T. aurantiacus* could produce a wide range of enzymes, including esters, alcohols, and other flavors, that contribute to the flavor of the liquor, adding a unique aroma and taste to the liquor (Tao et al., 2023). Therefore, from the point of view of microbial diversity, the control of microflora abundance during the maturation period of Qupi is crucial for the next step of baijiu fermentation.

The composition and abundance of dominant microorganisms significantly Influenced the formation and content of downstream volatiles. A total of 248 species and 11 major classes of compounds were detected, with alcohols being the most dominant flavor compounds, followed by esters and aldehydes. The volatile substances changed little during the maturation period, indicating that the volatile substances in Qupi tended to be balanced and stabilized. The flavor profile of Fengxiangxing baijiu was closely related to the content of esters, such as ethyl acetate, ethyl lactate, ethyl caproate, and ethyl butyrate, as well as the skeletal components of alcohols, such as isoamyl alcohol and n-propyl alcohol. Some substances related to the skeleton components of Fengxiangxing baijiu were detected at different stages. Among them, 1-butanol-3-methyl- had the highest average relative content in quatrefoil, followed by hydrazinecarboxamide, ethanol, and phenylethyl alcohol. 1-butanol-3-methyl- was the main component of alcohols in the FHD and was also the most abundant volatile compound contributing to malt and alcohol odors. Additionally, 1-butanol-3-methyl- can enhance the mellowness of the liquor, bringing unpleasant flavors and incongruities, such as bitterness and astringency, which may disrupt the equilibrium of the liquor in baijiu (Xu et al., 2023). Phenylethyl alcohol, 1-octen-3-ol, and (E)-2-octen-1-ol were also found in higher levels in the samples, which are mainly formed by the catabolism of amino acids through the Ehrlich metabolic pathway or lipid oxidation in certain microbial strains (Zhang et al., 2021). Phenylethyl alcohol has a honey aroma and is one of the main substances presenting the typical stylistic characteristics of Fengxiangxing baijiu (Liu et al., 2022). Ethyl acetate, ethyl hexanoate are the key esters of Fengxiangxing baijiu. In this study, the average relative content of ethyl acetate was higher than that of hexanoic acid and ethyl ester. Heterocyclic compounds, including pyrazine, furan, and indole, are the flavor substances that are most affected by the change in microbial community abundance during Daqu preparation. Among them, pyrazine is an important volatile compound in Chinese liquor, providing baking, burnt, and nutty aromas. Pyrazine has also been reported to be the key active compound in Jiangxiangxing baijiu (Yang L. et al., 2021; Yang Y. et al., 2021). The content of pyrazines in Fengxiangxing baijiu was lower than that of Jiangxiangxing baijiu. A total of 10 pyrazines were identified in this study, which were mainly present at the high-temperature and maturation period, with a relatively high content of 2,3,5,6-tetramethylpyrazine, and these pyrazines were differently distributed in Nongxiangxing, Jiangxiangxing, and Qingxiangxing baijiu, respectively (He et al., 2022; Dong et al., 2024; Yu et al., 2024). *Bacillus*, *Thermoactinomyces*, and *Aspergillus* are the major contributors of pyrazines (Dong et al., 2024). Additionally, the off-flavors produced by earth flavors have an important impact on the flavor quality of baijiu, especially the high concentration of earth flavors can bring obvious off-flavors to baijiu. *Streptomyces* and *Actinomyces* in the Qupi have been proven to be the main biological sources of earthy flavors in baijiu through culturable validation, while *Aspergillus* and *Bacillus* have strong earthy flavor inhibitory ability (Du et al., 2011, 2015). In this study, the earthy flavors were mainly produced during the high-temperature and maturation periods. Therefore, the higher abundance of *Aspergillus* and *Bacillus* detected during FHD preparation can be used as potential earthy flavor inhibitory strains to regulate the structure of the functional community of Daqu more accurately and improve the quality of baijiu.

In conclusion, significant correlations were found between the primary dominant microorganisms during the forming FHD from Qupi and the fermentation characteristics and flavor quality of Qupi (*p* < 0.05). During the warming period, the changes in the relative abundance of the dominant microorganisms, such as *F. sanfranciscensis*, *L. plantarum*, *S. fibuligera*, and *W. anomalus*, promoted the esterifying power and liquefying power as well as the formation of esters. During the high-temperature period, the changes in the relative abundance of dominant microorganisms, such as *B. licheniformis, F. sanfranciscensis, T. aurantiacus*, and *S. fibuligera*, increased the acidity value of Qupi and promoted the esterifying power, liquefying power, fermenting power, and the formation of acids. During the maturation period, the changes in the relative abundance of dominant microorganisms, such as *B. licheniformis, S. rectivirgula, T. aurantiacus*, and *A. amstelodami*, were associated with the esterifying power, liquefying power, saccharifying power and the accumulation of alcohols, aromatic compounds, and aldehydes. Moreover, the correlation analysis showed that different genera may have opposite correlations with the fermentation characteristics and volatile contents, suggesting that these genera may have diametrically opposed functional attributes to each other. Therefore, the microbial community composition during the forming FHD from Qupi is an issue of concern for improving the quality of Daqu, demanding future studies to investigate the metabolic mechanisms, interactions, and competitive and synergistic roles of different dominant genera in Daqu for high-quality Daqu production.

## 5 Conclusion

In summary, the changes in the sensory characteristics, fermentation characteristics, and flavor substances during the forming FHD from Qupi were mediated by the changes in microbial community structure. The results indicated that the 12th day of Qupi cultivation was the key process point of the microbial community structure succession, showing the most significant difference in microbial community structure sensory characteristics, fermentation characteristics, and flavor substances before and after the process, Eight microbial species were abundant in Qupi, but *B. licheniformis* (43.25%), *S. rectivirgula* (35.05%), *T. aurantiacus* (76.51%), *A. amstelodami* (10.81%), and *S. fibuligera* (8.88%) were the dominant species in FHD (CQ8). *S. fibuligera*, *A. amstelodami,* and *T. aurantiacus* had a greater influence on the sensory color of the epidermis and interior of FHD. The higher the abundance of *T. aurantiacus*, *A. amstelodami*, *B. licheniformis,* and *S. rectivirgula*, the higher the esterification and liquefying power of FHD. The higher the abundance of *T. aurantiacus* and *A. amstelodami*, the higher the saccharifying power of FHD. The higher the abundance of *S. fibuligera*, the stronger the fermenting power of FHD. The higher the abundance of *B. licheniformis*, *S. rectivirgula*, *T. aurantiacus,* and *S. fibuligera*, the higher the content of alcohols, aromatic compounds, and phenolics in the FHD. The higher the abundance of *S. fibuligera*, the higher the content of acids, esters, and hydrocarbons in FHD. This study is of great theoretical significance and application value for controllably adjusting the microbial colony structure of FHD, stably controlling the quality of FHD, and guiding the production of FHD in a precise, effective, and large-scale manner.

## Data availability statement

The original contributions presented in the study are included in the article/supplementary material, further inquiries can be directed to the corresponding author.

## Author contributions

DC: Writing – original draft, Writing – review & editing. JL: Writing – original draft, Writing – review & editing. JC: Investigation, Validation, Writing – review & editing. SX: Conceptualization, Resources, Writing – review & editing. CJ: Data curation, Investigation, Writing – review & editing. YoZ: Resources, Writing – review & editing, Validation. YuZ: Project administration, Writing – review & editing. WZ: Resources, Supervision, Writing – review & editing. JK: Project administration, Writing – review & editing.

## Footnotes

^1^https://cloud.majorbio.com

^2^http://huttenhower.sph.harvard.edu/LEfSe

^3^http://www.hmdb.ca/

^4^http://www.genome.jp/kegg/
